# The Impact of Volatile Anesthetic Choice on Postoperative Outcomes of Cardiac Surgery: A Meta-Analysis

**DOI:** 10.1155/2017/7073401

**Published:** 2017-08-29

**Authors:** Andres Zorrilla-Vaca, Rafael A. Núñez-Patiño, Valentina Torres, Yudy Salazar-Gomez

**Affiliations:** ^1^Faculty of Health, Universidad del Valle, School of Medicine, Hospital Universitario del Valle, Cali, Colombia; ^2^Department of Anesthesiology and Critical Care Medicine, The Johns Hopkins Hospital, Baltimore, MD, USA; ^3^Faculty of Health Sciences, Pontificia Universidad Javeriana, Cali, Colombia; ^4^Faculty of Health, Universidad Libre, Cali, Colombia

## Abstract

**Objectives:**

To evaluate the impact of volatile anesthetic choice on clinically relevant outcomes of patients undergoing cardiac surgery.

**Methods:**

Major databases were systematically searched for randomized controlled trials (RCTs) comparing volatile anesthetics (isoflurane versus sevoflurane) in cardiac surgery. Study-level characteristics, intraoperative events, and postoperative outcomes were extracted from the articles.

**Results:**

Sixteen RCTs involving 961 patients were included in this meta-analysis. There were no significant differences between both anesthetics in terms of intensive care unit length of stay (SMD −0.07, 95% CI −0.38 to 0.24, *P* = 0.66), hospital length of stay (SMD 0.06, 95% CI −0.33 to 0.45, *P* = 0.76), time to extubation (SMD 0.29, 95% CI −0.08 to 0.65, *P* = 0.12), S100*β* (at the end of surgery: SMD 0.08, 95% CI −0.33 to 0.49, *P* = 0.71; 24 hours after surgery: SMD 0.21, 95% CI −0.23 to 0.65, *P* = 0.34), or troponin (at the end of surgery: SMD −1.13, 95% CI −2.39 to 0.13, *P* = 0.08; 24 hours after surgery: SMD 0.74, 95% CI −0.15 to 1.62, *P* = 0.10). CK-MB was shown to be significantly increased when using isoflurane instead of sevoflurane (SMD 2.16, 95% CI 0.57 to 3.74, *P* = 0.008).

**Conclusions:**

The volatile anesthetic choice has no significant impact on postoperative outcomes of patients undergoing cardiac surgery.

## 1. Introduction

Multiple studies have shown the potential benefits of volatile anesthetics over the intravenous anesthetics in cardiac surgery [1]. Several meta-analyses have demonstrated that patients anesthetized with volatile anesthetics tend to have lower postoperative levels of troponin as compared with intravenous anesthesia [2–5], and consequently evidenced cardioprotective effects result in a decreased morbidity (i.e., reductions of myocardial infarctions, intensive care unit and hospital stay, time on mechanical ventilation, and incidence of long-term cardiac events) and mortality [6]. These benefits have also been extrapolated in noncardiac surgery according to a recent meta-analysis [7].

There are several opinions regarding what is the preferred volatile anesthetic. It is known that the newer drug sevoflurane has some chemical advantages over isoflurane (e.g., less solubility resulting in a faster onset and offset of action, less irritating to the airway, and being not as pungent as isoflurane; thus it can be used for inhalational induction of anesthesia [1, 6]. Moreover, there appears to be an opinion among cardiac anesthesiologists that sevoflurane is superior to isoflurane) [8]. However, there is still no a definite response if there is a volatile anesthetic with the best profile, neither a consensus nor recommendation about this topic. We undertook this meta-analysis comparing volatile anesthetics (isoflurane versus sevoflurane) in cardiac surgery, with the aim of evaluating the impact of volatile anesthetic choice on clinically relevant outcomes of patients undergoing cardiac surgery.

## 2. Methodology

This meta-analysis was performed according to Preferred Reporting Items for Systematic Review and Meta-Analyses (PRISMA) and following the recommendations of the Cochrane Collaboration [24, 25].

### 2.1. Eligibility Criteria

#### 2.1.1. Types of Studies

Two authors reviewed the literature and screened the abstracts independently. They selected all relevant articles in full text for detailed comprehension and further assessment of the quality and agreement of inclusion criteria. This meta-analysis focused on randomized controlled trials (RCTs). We did not restrict our selection criteria to studies developed at specific regions nor studies with very low sample size.

#### 2.1.2. Types of Participants

The participants included in this meta-analysis were adult patients (age > 18 years) who underwent cardiac surgery with either isoflurane or sevoflurane anesthesia.

#### 2.1.3. Types of Intervention

The use of isoflurane for maintenance of general anesthesia was considered the intervention in this meta-analysis, and the control group consisted of sevoflurane anesthesia.

#### 2.1.4. Types of Outcome Measures

The primary outcomes in this meta-analysis were the intensive care (ICU) length of stay and hospital length of stay. Secondary outcomes included the time to extubation, S100*β*, CK-MB, and troponin at the end of surgery and 24 hours after surgery.

### 2.2. Inclusion Criteria

- RCTs compared sevoflurane with isoflurane used for maintenance anesthesia in cardiac anesthesia.
- RCTs should recruit adult patients (age > 18 years) undergoing cardiac surgery (on-pump and off-pump).

### 2.3. Exclusion Criteria

We excluded studies that compared volatile anesthesia with nonvolatile anesthesia.

### 2.4. Literature Search

The MEDLINE/PubMed (from 1950 to Feb 2017), Google Scholar (from 1960 to Feb 2017), EMBASE (from 1980 to Feb 2017), and Cochrane library (from 1990 to Feb 2017) were searched for randomized controlled trials (RCTs) comparing the effects of isoflurane with sevoflurane anesthesia in cardiac surgery.

### 2.5. Search Strategy

The terms included in the search strategy were “cardiac surgery”, “volatile anesthesia”, “isoflurane”, “sevoflurane”, “troponin”, “hospital stay”, “intensive care unit stay”, and “randomized controlled trial”. We did not restrict for language. In addition, we reviewed citations of included articles in order to ensure inclusion of relevant studies not captured in our initial literature search.

### 2.6. Data Extraction and Management

Two authors verified and extracted the data of the eligible articles. They completed a predefined database in Excel that contained all the possibly relevant variables for this meta-analysis (year of publication, sample size, mean age, anesthetic regimen, type of surgery, and the outcomes).

### 2.7. Assessment of Methodological Quality

Two authors performed the methodological quality assessment and no disagreement arose. The quality of each study included in this meta-analysis was assessed by the Cochrane review criteria for randomized studies. The score was calculated for each study based on seven items (random sequence generation, allocation concealment, blinding of personnel who performed anesthesia, blinding of outcome assessment, incomplete outcome data, selective reporting, and other bias). Each item was scored between 2 and 0 (being 2 “positive,” 1 “unclear,” and 0 “negative”).

### 2.8. Statistical Analysis

First, an exploratory qualitative analysis was conducted to describe the characteristics of the studies included in this meta-analysis. The meta-analysis was performed using the Review Manager 5.3 (Cochrane Collaboration, Oxford, UK) with random-effect model (DerSimonian & Laird method) [26]. The duration of analgesia, pain scores and sensory and motor block duration, and opioid consumption were extracted as continuous variables and compared using standardized mean difference (SMD) with their respective 95% confidence intervals (CI). We used forest plots to illustrate the estimations and overall effect sizes (pooled SMD represented as a solid diamond at the bottom of the forest plot). PONV was extracted as a dichotomous outcome (present or absent) and compared using risk ratios (RRs). Heterogeneity of each meta-analyzed value was assessed by (*I*^2^) with the correspondent chi-squared test (*I*^2^ < 50% and *I*^2^ > 50% were considered insignificant and significant heterogeneity, resp.). Publication bias was calculated using Stata version 13.0 (Stata, College Station, TX) with Begg's and Egger's test [27]. Funnel plots were constructed to represent any tendency for publishing in favour of the positive effect. Significant publication bias was considered when there was asymmetry in the funnel plot (meaning that smaller studies tend to show larger SMDs). *P* values < 0.05 were considered as statistically significant in all statistical analyses.

## 3. Results

### 3.1. Literature Search Results

**Figure 1:**
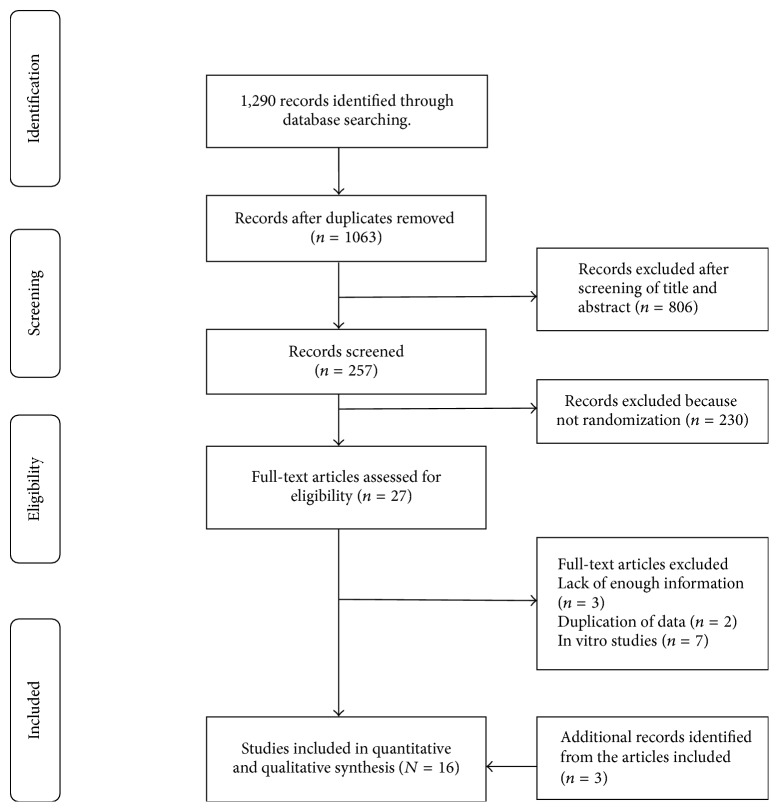
PRISMA flow chart of the selection of studies.

In total sixteen RCTs were included in the meta-analysis. The trial flow diagram illustrates the number of excluded and included articles in detail (Figure 1).

### 3.2. Study Characteristics

Sixteen potentially eligible articles were reviewed [8–23]. All the studies were published as original articles.  Table 1 summarizes the characteristics of the studies included in the analysis.

### 3.3. Meta-Analysis

**Figure 2:**
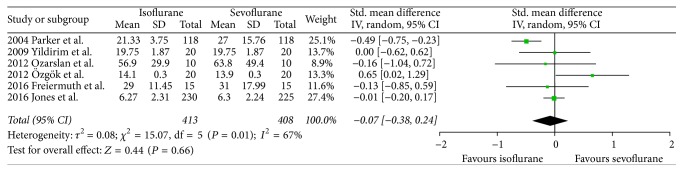
Forest plot comparing ICU length of stay between isoflurane and sevoflurane.

**Figure 3:**
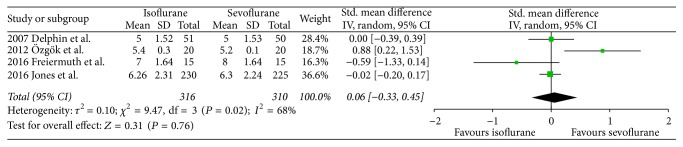
Forest plot comparing hospital length of stay between isoflurane and sevoflurane.

**Figure 4:**
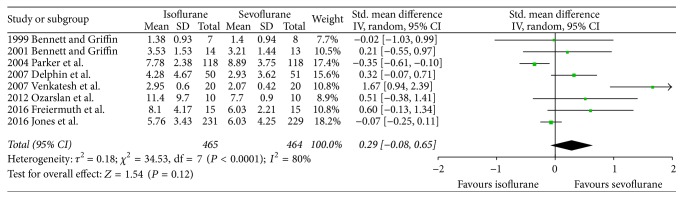
Forest plot comparing time of extubation between isoflurane and sevoflurane.

**Figure 5:**
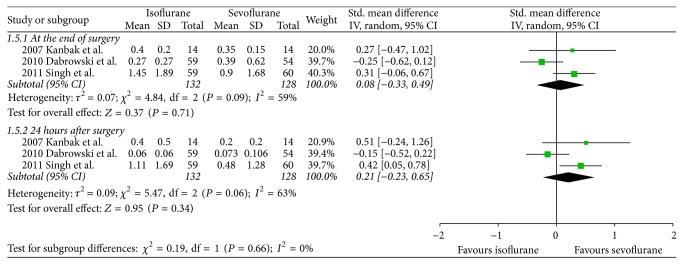
Forest plot comparing S100b between isoflurane and sevoflurane at the end of surgery and 24 hours after surgery.

**Figure 6:**
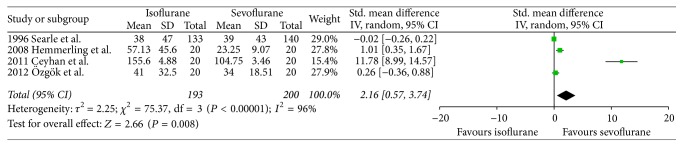
Forest plot comparing CK-MB between isoflurane and sevoflurane at 24 hours after surgery.

**Figure 7:**
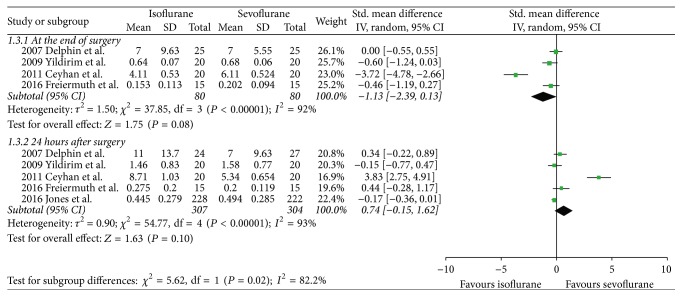
Forest plot comparing postoperative troponin levels between isoflurane and sevoflurane at the end of surgery and 24 hours after surgery.

There were no significant differences between both volatile anesthetics in terms of intensive care unit length of stay (SMD −0.07, 95% CI −0.38 to 0.24, *P* = 0.66; Figure 2), hospital length of stay (SMD 0.06, 95% CI −0.33 to 0.45, *P* = 0.76; Figure 3), time to extubation (SMD 0.29, 95% CI −0.08 to 0.65, *P* = 0.12; Figure 4), S100*β* (at the end of surgery: SMD 0.08, 95% CI −0.33 to 0.49, *P* = 0.71; 24 hours after surgery: SMD 0.21, 95% CI −0.23 to 0.65, *P* = 0.34; Figure 5), or troponin (at the end of surgery: SMD −1.13, 95% CI −2.39 to 0.13, *P* = 0.08; 24 hours after surgery: SMD 0.74, 95% CI −0.15 to 1.62, *P* = 0.10; Figure 6). CK-MB was shown to be significantly increased when using isoflurane instead of sevoflurane (SMD 2.16, 95% CI 0.57 to 3.74, *P* = 0.008; Figure 7); however this result was strongly influenced by only one study. Subgroup analysis by type of surgery (CABG or valvular surgery) showed no differences; there was only one study that included valvular surgery [11]. Therefore, the results did not change significantly.

### 3.4. Publication Bias

**Figure 8:**
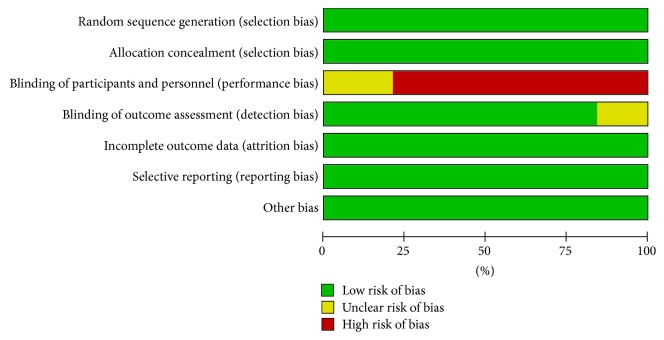
Risk of bias for each item.

Funnel plots were conducted to assess the publication bias in this meta-analysis of included studies. As shown in Supplemental File in Supplementary Material available online at https://doi.org/10.1155/2017/7073401, there was no evident asymmetry in the funnel plots. Therefore, the result suggested a low probability of publication bias. The quality assessment criteria ranged from 13 to 7 points for evidence synthesis (Figure 8).

## 4. Discussion

In this study, the use of isoflurane and sevoflurane was analyzed to obtain powerful conclusions regarding their outcomes in cardiac surgery. To our knowledge, this is the first meta-analysis providing a comparison between two inhaled anesthetics in patients undergoing cardiac surgery, showing that the difference was not statistically significant between the use of isoflurane and sevoflurane.

In recent years there have been several studies comparing anesthetics used in cardiac surgery because the latter represents a remarkably cause of morbidity and mortality worldwide, especially in developing countries [19, 28, 29]. Importantly, in cardiac anesthesia volatile anesthetics it is still one of the most important pharmacologic resources for anesthesia maintenance; it is because this type of anesthesia has a better profile (cardioprotective and neuroprotective) than nonvolatile anesthesia. In this scenario, some authors have hypothetically considered the implication of specific inhaled anesthetics in perioperative and postoperative complications. In terms of the properties of volatile anesthetics, relative blood/gas solubility isoflurane is higher than sevoflurane (1.38 and 0.66, resp.); therefore the last has the longer half-life in plasma; and the estimated minimum alveolar concentration (MAC) is the same in both anesthetics (1.15% and 2.05% per hour correspondingly) [22, 30]. Sevoflurane generally costs the most and isoflurane the least. Nevertheless, it is difficult to estimate the precise cost of each inhaled anesthetic due to sharing of devices used to vaporize the medication and the individual dosage according to the weight of each individual [31]. In summary, although the general properties of individual inhaled anesthetics are different, there is not a preferred volatile anesthetic so far in clinical practice.

The overall results of the randomized clinical trials included in this study did not show statistically significant difference between the use of isoflurane and sevoflurane in terms of the primary clinical outcomes ICU length of stay and time of extubation (SMD = −0.07; 95% CI = −0.43, 0.28; *P* = 0.66 and SMD = 0.29 95% IC = 0.08, 0.65; 95% CI; *P* = 0.18, resp.). Secondary clinical outcomes are discussed as follows. The difference of isoflurane and sevoflurane in hospital length of stay was not statistically significant (SMD = 0.06; 95% CI = −0.33, 0.45; *P* = 0.76). Neurological dysfunction is one of the complications associated in patients undergoing cardiopulmonary bypass (CPB) and occurs in 50–80% and may persist several months in 20–30% of these patients [13]. S100*β* is a nonspecific cerebral tissue protein commonly used in clinical research as a biomarker of neurological impairment, in the setting of cardiac surgery, when it crosses the blood-brain barrier through the bloodstream after glial damage [21]. It has been documented that volatile anesthetics would provide neuroprotection through different mechanisms, especially against cerebral ischemic injury [18, 21]. It has been documented that volatile anesthetics would provide neuroprotection, especially against cerebral ischemic injury, through different mechanisms such as inducible nitric oxide synthase (iNOS), mitochondrial K_ATP_ channels, and ubiquitin conjugated protein aggregation [18, 32–34]. Difference in levels of S100*β* using isoflurane or sevoflurane was not statistically significant (SMD = 0.08; 95% CI = −033 to 0.49; *P* = 0.71 and SMD = 0.21; CI 95% = −0.23, 0.65; *P* = 0.06, at the end of the surgery and 24 hours after the surgery, resp.). Volatile anesthetics have been shown to be cardioprotective from ischemic injury through different mechanisms, similar to neuroprotectives, such as key roles of K_ATP_ channels and adenosine A1 receptors, which improved coronary perfusion mediated by increased nitric oxide production [18, 21, 35, 36]. Difference was statistically significant favouring sevoflurane SMD = 2.16; 95% CI = 0.57, 3.74; *P* = 0.008. However, the number of included RCTs regarding the outcome cardiac troponin-T (cTnT) (*n* = 4) is small; the heterogeneity is substantial (*I*^2^ = 96%) and a single study was influencing the pooled results [19]. cTnT is part of the contractile apparatus in myofibrils [37] and is used as an indicator of the severity of damage after cardiac surgery [37]. Levels of TnT were also analyzed; the difference between the two inhaled anesthetics was not statistically significant at the end of the surgery (SMD = −1.13; 95% CI = −2.39, 0.13; *P* = 0.08) and 24 hours after surgery (SMD = 0.74; 95% CI = −0.15, 1.62; *P* = 0.10).

We consider that this study follows a comprehensive retrospective analysis of the included RCTs and exhaustive assessment of the identified primary clinical outcomes. The systematic search in major databases was wide and exhaustive and the results are consistent. Quality analysis of this study did not evidence substantial publication bias. However, this meta-analysis has limitations, listed as follows. High heterogeneity was detected comparing the difference between both anesthetics for primary clinical outcomes: time of extubation and ICU length of stay (*I*^2^ = 80%; *P* for heterogeneity < 0.01 and *I*^2^ = 67%; *P* for heterogeneity = 0.01, resp.). Substantial heterogeneity was also found in secondary clinical outcomes such as Hospital LOS (*I*^2^ = 68%; *P* for heterogeneity = 0.02), CK-MB levels (*I*^2^ = 96%; *P* for heterogeneity < 0.01), and troponin levels (*I*^2^ = 82.2%; *P* for heterogeneity = 0.02). In addition, when aiming to estimate effect sizes, frequently a single study with small sample size was influencing the results from the pool of RCTs included. Nonetheless, larger RCTs were suggesting no significantly difference in the overall results. Furthermore, a small number of high-quality RCTs were included for the estimation of clear statistical parameters in clinical outcomes such as plasmatic levels of CK-MB and S100*β* in perioperative and postoperative instances.

## 5. Conclusions

The volatile anesthetic choice has no significant impact on postoperative outcomes of patients undergoing cardiac surgery. Other practical considerations (availability, costs, and preference) may be influential factors into the decision regarding which anesthetic to use.

## Supplementary Material

Funnel plots illustrating the publication bias for each outcome evaluated in this meta-analysis.

## Figures and Tables

**Table 1:** Characteristics of the studies included in this meta-analysis [8–22].

Study, reference	Study design	Type of cardiac surgery	Sample size	Anesthetic regimen	Outcomes	Conclusion
Searle et al. 1996 [9]	Multicentre, randomized, open-labelled study	CABG	Iso: 133 Sev: 140	Induction with midazolam (0.1–0.3 mg/kg) and fentanyl (5–15 *µ*g/kg), then a bolus of fentanyl 10 *µ*g/kg Iso: 2.3%; Sev: 4.1%. End-tidal concentrations of each anaesthetic vapor were kept less than 2.0 MAC equivalents	Myocardial infarction (assessed with CK-MB and ECG changes), ventricle failure, cardiac death, noncardiac death, and other hemodynamic events	Either Sev or Iso combined with fentanyl provided acceptable hemodynamic outcomes in patients with low risk who underwent elective CABG

Bennett and Griffin 1999 [10]	Prospective, crossover, dose-response study	CABG	Iso: 8 Sev: 8	Induction with midazolam (1-2 mg), fentanyl 3 *µ*g/kg, propofol as required and pancuronium 8 mg; then at sternotomy, fentanyl 4 *µ*g/kg and midazolam 1 mg MAC limits of 0.3 and 1.6 were used for Sev and Iso. In the crossover study, before surgery hemodynamic and cardiac profile were recorded at the following phases: (1) no volatile agent; (2) Iso 0.6%, Sev 0.9% (0.5 MAC); (3) Iso 1.2%, Sev 1.8%; (1.0 MAC); (4) no volatile agent; (5) Iso 0.6%, Sev 0.9% (0.5 MAC); (6) Iso 1.2, Sev 1.8 (1.0 MAC); (7) no volatile agent	Hemodynamic outcomes (HR, CI, SVRI, PVRI, SAP, PAP, CVP, and PCWP). Postoperative outcomes such as time of operation, time to open eyes, time of extubation, recall, memory, PONV, and general condition were also reported	Iso and Sev used as the primary anesthetic showed no statistical difference between them at any stage of the study

Bennett and Griffin 2001 [11]	Prospective, crossover, dose-response study	Valvular surgery	Iso: 14 Sev: 13	Induction with midazolam (1-2 mg), fentanyl 3 *µ*g/kg, propofol as required and pancuronium 8 mg; then at sternotomy, fentanyl 4 *µ*g/kg and midazolam 3 mg In the crossover study the protocol used was the same as that of Bennett and Griffin 1999	Same hemodynamic outcomes as measured in Bennett and Griffin 1999 [10]. Postoperative outcomes such as time of operation, time to open eyes, time of extubation, inotrope infusion, use of vasodilators, PONV, and memory were reported as well	Sev showed a tendency to lower heart rates and cardiac index compared with Iso. Nonetheless, these findings have shown no significantly difference

Parker et al. 2004 [12]	3-arm (isoflurane, sevoflurane, or propofol), randomized, controlled trial with patients and intensive care staff blinded to the drug allocation	CABG	Iso: 118 Sev: 118 Pro: 118	Induction with fentanyl 10 *µ*g/kg, diazepam 0.1 mg/kg, and pancuronium 0.15 mg/kg. Then fentanyl 5 *µ*g/kg two minutes before sternotomy. During surgery, isoflurane (end-tidal concentration 0.5% to 2%), sevoflurane (end-tidal concentration 1% to 4%), or propofol (target concentration 1–8 *µ*g/mL)	Time to extubation, ICU stay, and perioperative hemodynamics and perioperative drugs administered	Time to tracheal extubation was significantly longer for the target-controlled propofol group; however a significantly greater number of patients in this group required the use of a vasodilator to control intraoperative hypertension

Kanbak et al. 2007 [13]	Prospective and randomized study	CABG with CPB	Iso: 14 Sev: 14 Des: 14	Before CPB: Group Iso: 1% to 1.5%; Group Sev: 1.5% to 2%; Group Des: 7% to 8% During CPB: Group Iso: 0.5% to 1%; Group Sev: 1%; Group Des: 4% to 5%	Plasmatic levels of S100*β* in different operative instances and neuropsychological tests such as Minimental State Examination and Visual-Aura Digit Span Test	Iso was associated with better neurocognitive functions than Des or Sev after on-pump CABG. Sev seems to be associated with the worst cognitive outcome as assessed by neuropsychological tests, and prolonged brain injury as detected by high S100*β* levels was seen with Des

Delphin et al. 2007 [14]	Prospective and randomized trial	OPCAB	Iso: 50 Sev: 51	Volatile agents were titrated to maintain hemodynamic variables within 20% of their baseline values. Both groups received fentanyl 5 *µ*g/kg	Time variables after the surgery (including duration of anesthesia, duration of surgery, time to extubation, and hospital LOS). Neuropsychological scores and troponin enzyme levels after the surgery were also measured	Both Sev and Iso may be safely used as maintenance agents in OPCAB. Sev has the advantage of allowing earlier extubation and evaluation of neuropsychological tests after OPCAB

Venkatesh et al. 2007 [15]	Prospective and randomized trial	OPCAB	Iso: 20 Sev: 20	Induction with thiopentone sodium, midazolam (0.05–0.1 mg/kg) and fentanyl citrate (4 *μ*g/Kg). Groups with Sev or Iso 0.5–2% till end-tidal concentration of agent of 1.5–2%. Then fentanyl (50 *μ*g) and vecuronium (1 mg) were repeated at regular interval of 1 hour until the end of the surgery	Hemodynamic data (HR, MAP, PAP, CI, and others), depth of anesthesia, ischemic changes (assessed through blood CK-MB levels and ECG changes), time of awakening, and time of extubation	Both anesthetics are safe. Sev provides early awakening and extubation as compared with Iso

Yildirim et al. 2009 [16]	Prospective, randomized, and controlled trial	CABG with CPB	Iso: 20 Sev: 20 Pro: 20	Iso: induction: 1 *μ*g/kg bolus of remifentanil and midazolam 0.1 mg/kg, followed by a continuous infusion of 0.4 *μ*g/kg/min; maintenance: 0.3–0.6 *μ*g/kg/min of remifentanil and Iso 0.5%–1% Sev: induction: 1 *μ*g/kg bolus of remifentanil followed by a continuous infusion of 0.4 *μ*g/kg/min; Sev was started at 8% and when the patient was asleep lowered of 2%; maintenance: 0.3–0.6 *μ*g/kg per minute remifentanil and 0.5%–2% Sev Pro: induction: 1 *μ*g/kg bolus of remifentanil, followed by 0.4 *μ*g/kg/min and an infusion of propofol 2 *μ*g/mL; maintenance: 0.3–0.6 *μ*g/kg per minute remifentanil and 2–4 mg/mL infusion of propofol	Hemodynamic data (HR, MAP, PAP, CVP, PCWP, CO, CI, SVRI), myocardial oxidative stress status, and troponin I changes	Inhalation anesthetics preserved cardiac function in coronary surgery patients after CPB with less evidence for myocardial damage than propofol

Hemmerling et al. 2008 [17]	Prospective randomized double-blind trial	OPCAB	Iso: 20 Sev: 20	Induction with fentanyl 3 mg/kg, followed by propofol 1-2 mg/kg. 1 MAC for each volatile agent for maintenance	Arterial blood gases, peak expiratory flow, hemodynamic data, myocardial protection (measured by blood levels of CK-MB and troponin-T), left ventricular ejection fraction, postoperative pain, and time of extubation	Both volatile agents offer the same myocardial protection but Sev was associated with a shorter time to extubation

Singh et al. 2011 [18]	Prospective, randomized single-blinded trial	CABG with CPB	Iso: 59 Sev: 60 TIVA: 61	Induction: intravenous midazolam 2 mg, fentanyl 3–5 mg/kg, and thiopentone 3–5 mg/kg Maintenance: boluses of fentanyl and midazolam in each group. Iso: 1 MAC; Sev: 1 MAC; TIVA: fentanyl 4 *μ*g/kg/h, and midazolam 0.1 mg/kg/h	Hemodynamic data and S100*β* blood levels	S100*β* levels are diminished during Sev use in contrast to Iso and TIVA. The hemodynamic changes in the first 24 h do not seem to be influenced by these interventions

Ceyhan et al. 2011 [19]	Prospective and randomized trial	CABG with CPB	Iso: 20 Sev: 20	Induction: etomidate 0.3 mg/kg, a bolus dose of pancuronium 0.1 mg/kg, and remifentanil 1 *μ*g/kg was administered Maintenance: Sev: 2–4%; Iso: 1-2%. Both groups were started on a remifentanil infusion (0.1–0.4 *μ*g/kg/min)	Hemodynamic data. Troponin-T, CK, and CK-MB levels	Sev provides a better myocardial protection than Iso, with lower levels of troponin-T and CK-MB observed with Sev

Dabrowski et al. 2010 [21]	Prospective and randomized trial	CABG with CPB and ECC	Iso: 54 Sev: 59 No volatile: 66	Induction: fentanyl (0.01–0.02 mg/kg), midazolam (0.05–0.1 mg/kg), and etomidate (0.1–0.5 mg/kg) Maintenance: Iso: 0.5%–1%; Sev: 0.5%–1%	Hemodynamic data and S100*β* blood levels	After cardiac surgery S100*β* elevation was evidenced. Iso and Sev significantly reduced plasma S100*β* concentrations

Ozarslan et al. 2012 [20]	Prospective and randomized trial	CABG with CPB	Iso: 10 Sev: 10 Des: 10	Induction: etomidate 0.4 mg/kg, vecuronium bromide 0.1 mg/kg, and fentanyl, 1 *μ*g/kg Maintenance: Iso: 1%-2%; Sev: 2%-3%; Des: 4%–6%. All volatile agents were given at 1 MAC in an oxygen-air mixture, and remifentanil was at 0.025 mg/kg/min	Hemodynamic data, laboratory parameter (such as hematocrit, lactate and potassium), and microcirculatory parameters	Sev had a negative effect on the microcirculation. Iso decreased vascular density and increased flow. Des produced stable effects on the microcirculation. All inhalation agents induced transient alterations in microvascular perfusion

Özgök et al. 2012 [23]	Prospective and randomized trial	CABG with CPB	Iso: 20 Sev: 20	Induction: intravenous bolus infusion of midazolam (0.1 mg/kg), fentanyl (15–20 m/kg), and intravenous pancuronium bromide (0.1 mg/kg) Maintenance: doses of fentanyl 5 *μ*g/kg and pancuronium bromide 2 mg were applied repeatedly as required in this group. Sevoflurane or isoflurane was administrated in 1 MAC (minimal alveolar concentration)	Hemodynamic parameters, CK-MB, troponins, lactate	No significant differences between volatile agents

Freiermuth et al. 2016 [22]	Prospective and randomized trial	CABG with CPB and MECC	Iso: 15 Sev: 15	Induction: propofol 1-2 mg/kg, fentanyl 3–5 *μ*g/kg, and atracurium, 0.5 mg/kg Maintenance: propofol infusion 4–10 mg/kg/min. The vaporizer was set at a fixed fractional amount of Sev and Iso into the fresh gas supply of 1.8 and 0.8, respectively, at a flow rate of 2-3 L/min	Pharmacokinetics measurements, blood troponin levels, total dose of norepinephrine during MECC, intubation time, ICU LOS, hospital LOS, and mortality within 30 days	Similar pharmacokinetics regarding wash-in and wash-out for Sev and Iso. No significantly differences in cardiovascular stability and markers of cardiac damage were found

Jones et al. 2016 [8]	Pragmatic randomized noninferiority comparative effectiveness clinical trial	CABG, CPB, and/or single valve repair or replacement	Iso: 233 Sev: 231	Induction: fentanyl (5–10 *μ*g/kg) or sufentanil (1–5 *μ*g/kg), midazolam (0.05–0.1 mg/kg), propofol (0.25–1 mg/ kg^−1^), and rocuronium (0.6–1.2 mg/kg). Both volatile agents were administered at a dose of 0.5–2.0 MAC throughout the entire operation	ICU LOS, mortality, troponin T levels, ICU lengths of stay, duration of tracheal intubation, inotrope or vasopressor usage in the ICU, inotrope or vasopressor usage, peak postoperative serum creatinine, new-onset hemodialysis, new-onset atrial fibrillation, use of an intra-aortic balloon pump, perioperative stroke, and ICU readmission	Sev is noninferior to isoflurane on a composite outcome of prolonged ICU stay and mortality. Sev is not superior to Iso on any other of the clinically important outcomes

Iso: isoflurane; Sev: sevoflurane; Des: desflurane; Pro: propofol; CABG: coronary artery bypass graft; CPB: cardiopulmonary bypass; ECC: extracorporeal circulation; MECC: minimized extracorporeal circulation; OPCAB: off-pump coronary artery bypass; ECG: electrocardiogram; PONV: postoperative nausea and vomiting; ICU: intensive care unit; LOS: length of stay; CK: creatine kinase; CK-MB: creatine kinase-MB; TIVA: total intravenous anesthesia; *Hemodynamic Data*. HR: heart rate; MAP: mean arterial pressure; PAP: pulmonary artery pressure; CI: cardiac index; CO: cardiac output; MAC: minimum alveolar concentration; CVP: central venous pressure; PCWP: pulmonary capillary wedge pressure; SVRI: systemic vascular resistance index; SAP: systemic arterial pressure; PVRI: pulmonary vascular resistance index.
