# Supplementary material for: The Impact of Volatile Anesthetic Choice on Postoperative Outcomes of Cardiac Surgery: A Meta-Analysis
Source: Biomed Res Int. 2017 Aug 29;2017:7073401. doi: 10.1155/2017/7073401 (PMC5603325; doi:10.1155/2017/7073401)

SUPPLEMENTAL FILE

Funnel plot of figure 2

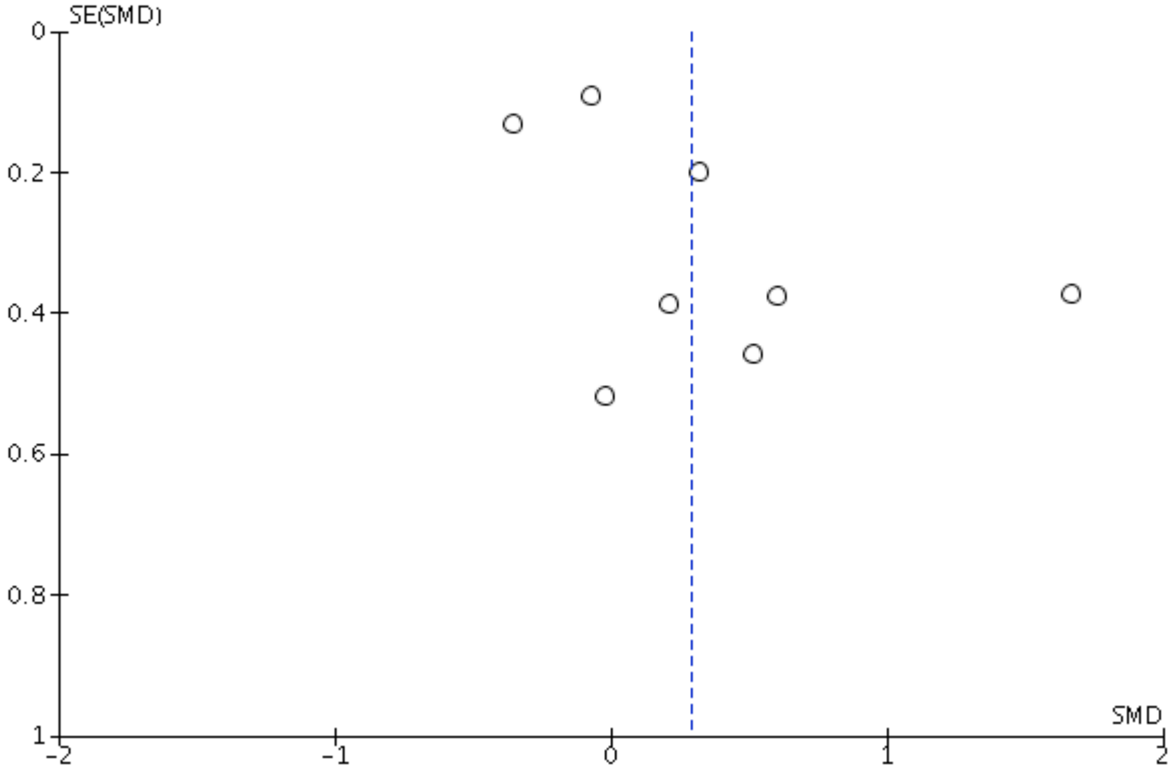

Funnel plot of figure 3

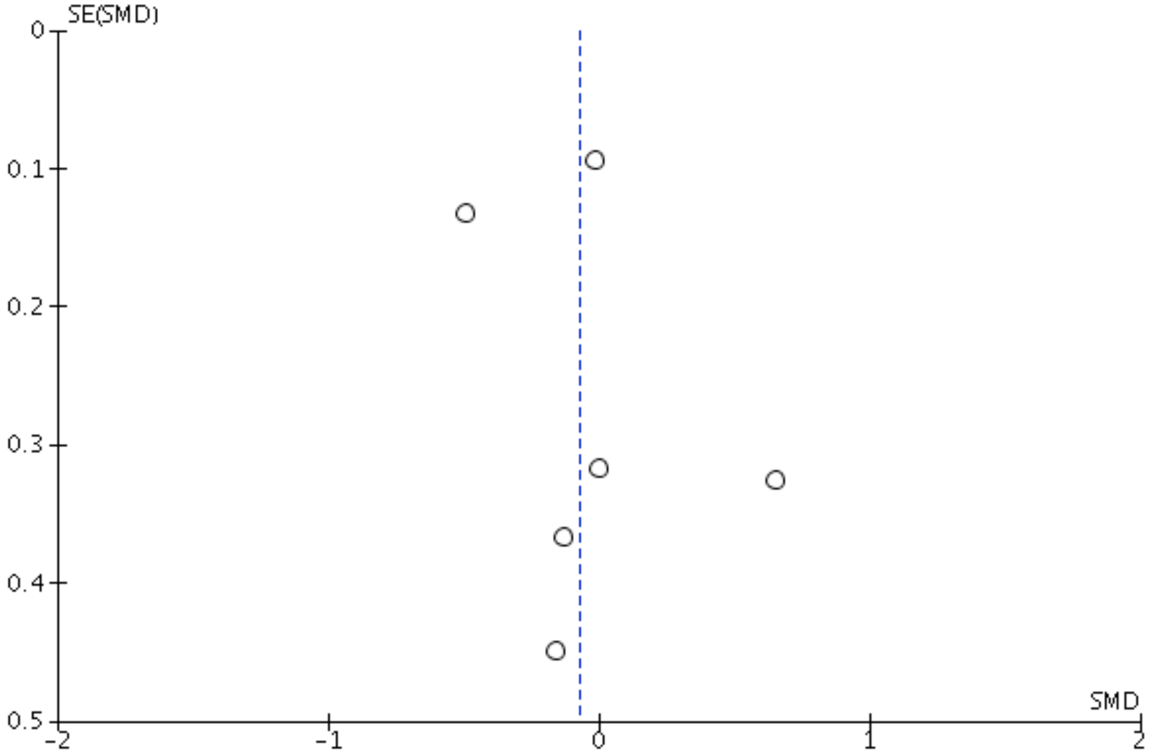

Funnel plot of figure 4

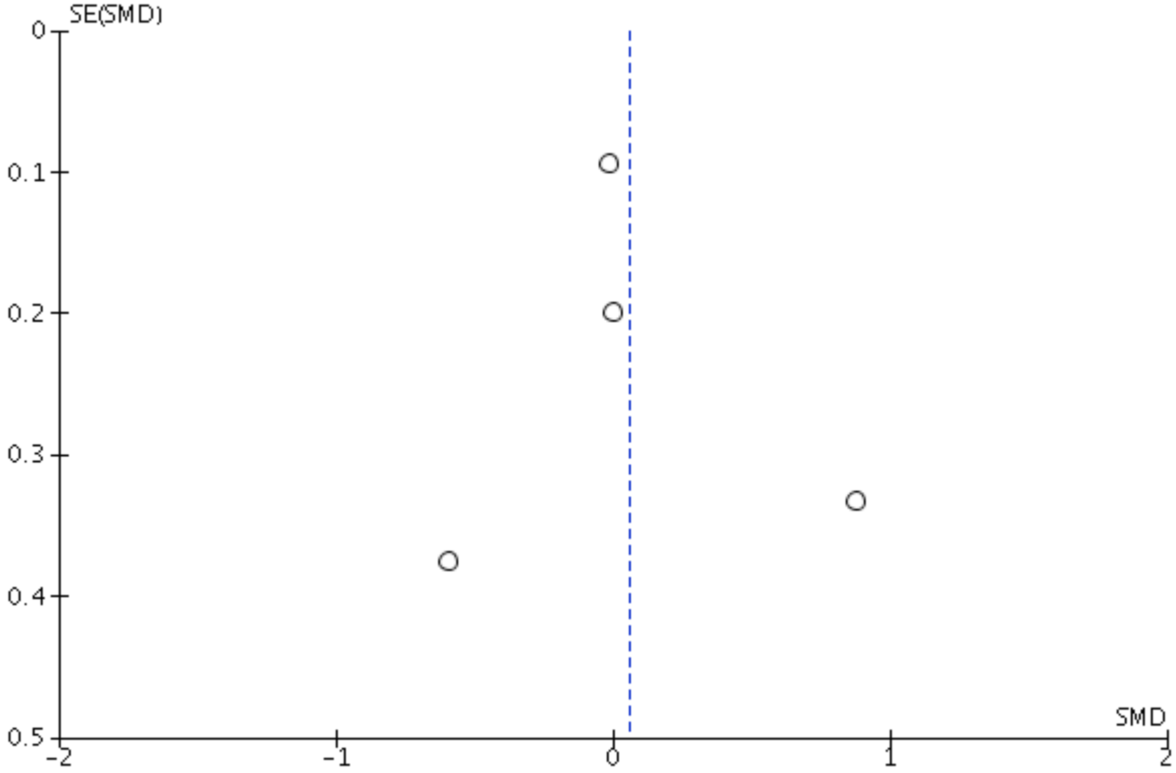

Funnel plot of figure 5

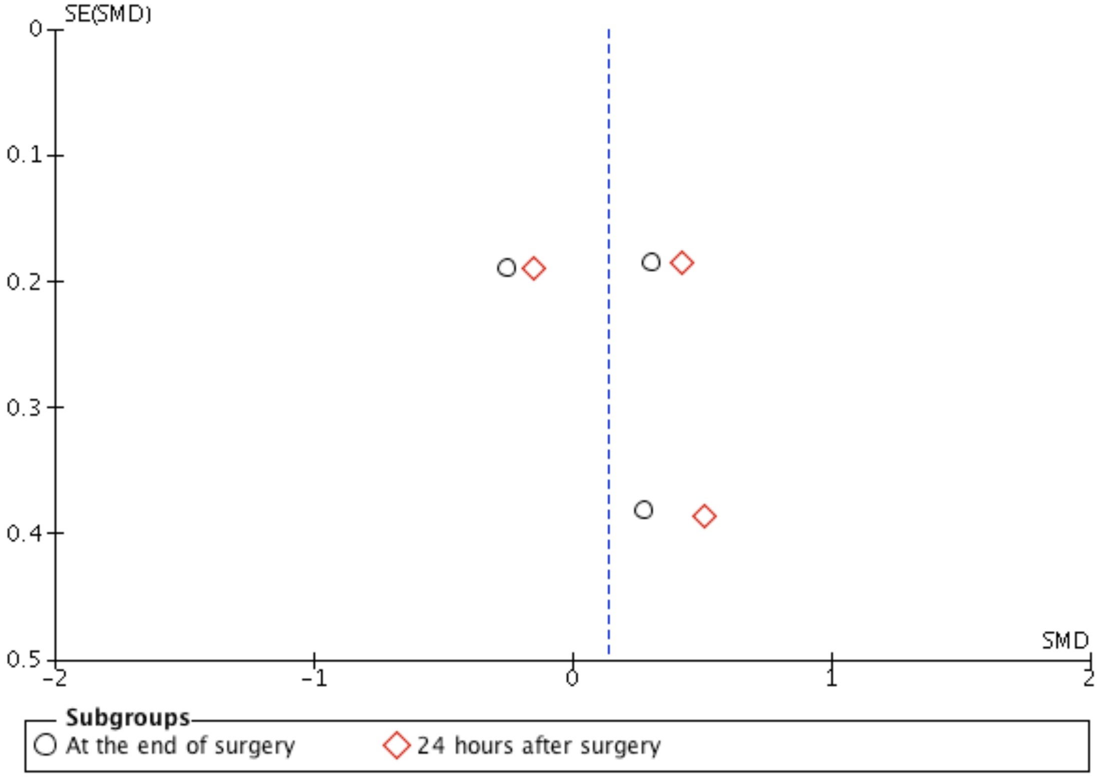

Funnel plot of figure 6

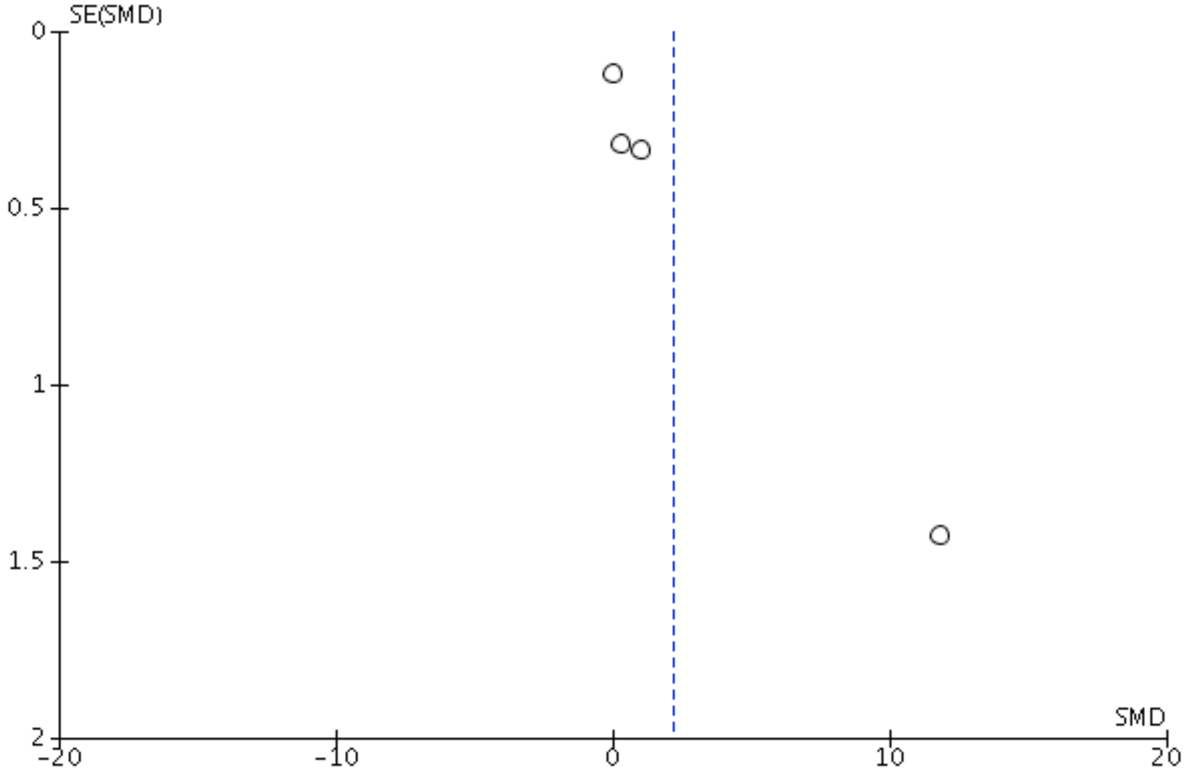

Funnel plot of figure 7

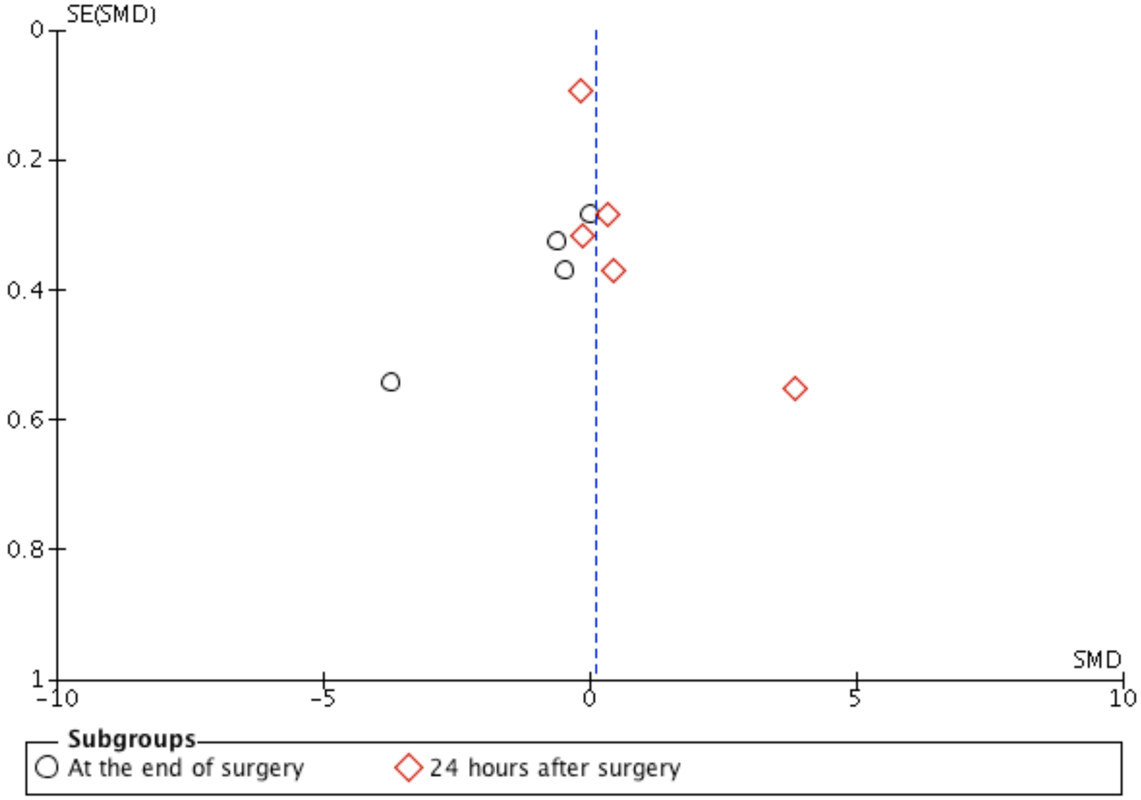

Supplement: Supplementary file 1 — Funnel plots illustrating the publication bias for each outcome evaluated in this meta-analysis. [file 7073401.f1.pdf]
